# Live Attenuated aTJ Vaccine Effectively Protects Pigeons Against Homologous PPMV-1 Challenge

**DOI:** 10.3390/vaccines12121304

**Published:** 2024-11-22

**Authors:** Shan Zhang, Dahu Liu, Baojing Liu, Ruinying Liang, Lin Liang, Xinming Tang, Shaohua Hou, Chan Ding, Xusheng Qiu, Jiabo Ding

**Affiliations:** 1 Institute of Animal Sciences, Chinese Academy of Agricultural Sciences, Beijing 100193, China; zhangshan0276@163.com (S.Z.); liudahu961196@163.com (D.L.); liangruiying@caas.cn (R.L.); lianglin@caas.cn (L.L.); tangxinming@caas.cn (X.T.); houshaohua@caas.cn (S.H.); 2 Beijing Xinhexiang Technology Co., LLC, Beijing 100085, China; lbjxhxbio@163.com; 3 Shanghai Veterinary Research Institute, Chinese Academy of Agricultural Sciences, Shanghai 200241, China; shoveldeen@shvri.ac.cn

**Keywords:** PPMV-1, reverse genetics, aTJ strain, booster immunization, vaccine efficacy

## Abstract

**Background:** Pigeon paramyxovirus type 1 (PPMV-1) is a significant pathogen affecting pigeon populations globally. The commonly used La Sota vaccine provides limited protection due to antigenic divergence from circulating PPMV-1 strains. An antigenically matched vaccine is needed to address this challenge. **Methods:** An attenuated aTJ strain was developed through reverse genetics by modifying the F protein cleavage site of the virulent TJ-WT strain. Pigeons were immunized twice with the aTJ strain via eyedrop and intranasal routes, followed by a challenge with a virulent PPMV-1 strain ten days after the booster immunization. **Results:** The attenuated aTJ strain induced robust serum antibody titers post-booster immunization, and vaccinated pigeons showed strong protection upon challenge, with significantly reduced morbidity, mortality, and viral shedding compared to controls. **Conclusions:** These findings suggest that the aTJ strain is a promising candidate for the promotion of PPMV-1 prevention and control, emphasizing the importance of antigenic matching in optimizing vaccine efficacy.

## 1. Introduction

Newcastle disease (ND), caused by Newcastle Disease Virus (NDV), is a highly contagious disease that affects a wide range of avian species and leads to considerable economic losses in the global poultry industry [1]. NDV, also known as Avian Paramyxovirus Type 1 (APMV-1), is classified under the genus *Orthoavulavirus* in the family *Paramyxoviridae* [2]. Its non-segmented, negative-sense RNA genome is approximately 15.2 kb in length and encodes six major structural proteins: nucleoprotein (NP), phosphoprotein (P), matrix protein (M), fusion protein (F), hemagglutinin-neuraminidase (HN), and large polymerase (L). This genome follows the “rule of six” for genome length, a key feature in the replication process of paramyxoviruses [3]. Pigeon Newcastle Disease Virus, also named Pigeon Paramyxovirus-1 (PPMV-1), is a host-specific variant of NDV and predominantly falls within genotype VI of Class II. The PPMV-1 genome is approximately 15,192 nucleotides in length. First identified in Middle Eastern pigeons in 1978 [4], PPMV-1 contributed to the third ND panzootic in the 1980s [5]. Pigeons of all ages are susceptible to this virus, with the morbidity and mortality rates exceeding 50% [6]. Common clinical signs in infected pigeons include neurological symptoms such as paralysis, torticollis, and twisted neck, with the neurotropic, acute form being the most prevalent [7,8]. The disease was first reported in China in 1985, and it continues to pose a significant threat to both the pigeon breeding industry and wild bird populations [4].

Although commercial vaccination programs have been employed to control ND in poultry, current vaccines like the La Sota strain (genotype II), have demonstrated limited efficacy in pigeons [9,10]. Pigeons often display high viral shedding and morbidity due to inadequate immune protection, primarily caused by substantial antigenic differences between pigeon NDV strains (Class II, genotype VI) and the chicken-derived vaccine strains [11]. Pigeon farming, including both meat production and racing pigeon breeding, has grown rapidly due to its low costs and high economic value. In China, approximately 40 million pairs of breeding pigeons exist, with around 700 million pigeons sold for meat annually [12]. Additionally, the global racing pigeon industry is thriving, with competitions and high-value auctions driving economic growth [13,14]. As pigeon farming becomes more large-scale and intensive, the frequent introduction of breeding pigeons and increased interregional movement heightens the risk of disease transmission, posing significant challenges to disease control [12,15]. While the pathogenicity of PPMV-1 varies among different host species, several studies have shown that its virulence increases with subsequent passages through chickens [16,17]. The extensive flight range of pigeons facilitates the broader spread of PPMV-1, with prolonged viral shedding contaminating the environment and raising the risk of cross-species transmission [14]. Notably, PPMV-1 has been linked to fatal respiratory diseases in immunocompromised human patients, raising significant public health concerns [18]. These factors highlight the urgent need for pigeon-specific ND vaccines tailored to genotype VI’s unique antigenic properties, which could significantly improve pigeon immune protection and ensure the healthy development of the pigeon farming industry. Numerous studies have substantiated that vaccines genotypically aligned with epidemic strains markedly enhance ND control by diminishing viral shedding among affected birds [19,20,21]. While using inactivated epidemic strains as vaccines offers a strategic approach [13,22], developing these vaccines from virulent NDV strains requires strict operational and laboratory protocols. On the other hand, live attenuated NDV vaccines are well suited for mass production and are commonly used for routine large-scale vaccination through spray or drinking water administration [23]. Understanding the pathogenicity of NDV is crucial for vaccine development, as it is closely associated with the amino acid sequence of the F protein cleavage site (Fcs). Virulent viruses typically possess a polybasic amino acid motif of _112_R/KRQK/RRF_117_, whereas lentogenic viruses feature a monobasic amino acid sequence of _112_G/EK/RQG/ER-L_117_ [24,25,26]. The Fcs of PPMV-1 is commonly _112_RRQKRF_117_, which is characteristic of virulent strains [27]. Research using reverse genetics has demonstrated that replacing the Fcs of the La Sota strain with that of a virulent NDV strain results in increased virulence [28]. Conversely, modifying the Fcs of a highly virulent NDV to match the La Sota strain significantly reduces its virulence while preserving immunogenicity, making the recombinant virus a strong candidate for vaccine development [20,29]. These findings have laid the foundation for the use of attenuated viruses in ND vaccine development [20].

In this study, we employed a reverse genetics platform to generate an attenuated pigeon NDV strain by introducing mutations at the Fcs to match the La Sota sequence. Immunization of pigeons with the rescued strain, followed by booster immunization, resulted in robust serum antibody titers and significant protection against PPMV-1, including reduced morbidity, mortality, and viral shedding. These findings demonstrate that the attenuated strain not only holds promise as an effective vaccine candidate for controlling ND in pigeons but also offers a feasible approach to enhance biosecurity and reduce the risk of cross-species transmission.

## 2. Materials and Methods

### 2.1. Ethics Statement

The protocol involving animals was approved by the Animal Welfare and Ethics Committee of the Institute of Animal Sciences (IAS), Chinese Academy of Agricultural Sciences (Approval No. ISA2023-120, CAAS, Beijing, China).

### 2.2. Virus, Cells, and Plasmids

Pigeon paramyxovirus-1 Pigeon/TJ/CH/020/2020(TJ-WT) strain, GenBank PQ563353, was isolated from pigeons in China by our laboratory and identified as the dominant epidemic strain of subtype VI.2.1.1.2.2 [30]. TJ-WT was propagated in 9-day-old specific pathogen-free (SPF) chicken embryos (Beijing Boehringer Ingelheim Vital Bio, Beijing, China). Baby hamster kidney fibroblast cells (BHK21) were from the Cell Resource Center, Peking Union Medical College. BSR-T7/5 cells were kindly provided by Zhigao Bu and maintained in our laboratory for further experiments. The pLaSota-DM were kept in our laboratory [31]. pCI-neo plasmid was purchased from Miao Ling biotechnology (Shanghai, China).

### 2.3. Construction of Infectious cDNA Clones

**Figure 1:**
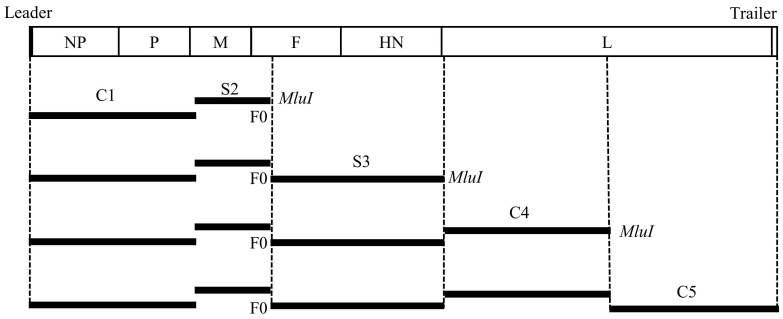
Schematic representation of the construction of the full-length genome plasmid TVT-aTJ for reverse genetics of the TJ-WT strain. The TJ-WT genome was divided into five segments (C1, S2, S3, C4, and C5) for sequential ligation into the pTVT vector using homologous recombination. MluI restriction enzyme digestion was employed to facilitate the insertion of the S3, C4, and C5 fragments. The label “F0” indicates the cleavage site of the F protein (Fcs), which was replaced with that of the La Sota strain. This process resulted in the construction of the full-length infectious clone, TVT-aTJ.

Viral RNA was extracted from the allantoic fluid infected with the TJ-WT strain by the FastPure Viral DNA/RNA Mini Kit (Vazyme Biotech, Nanjing, China). First-strand complementary DNA (cDNA) was synthesized using the Hifair III 1st Strand cDNA Synthesis Kit (YEASEN, Shanghai, China). To obtain the full genome sequence of the TJ-WT strain, cDNA was used as a template for PCR amplification with 11 pairs of primers [32]. The amplification was performed using PrimeSTAR MAX DNA Polymerase (Takara, Kyoto, Japan), according to the manufacturer’s instructions. All PCR products were sequenced by the Sanger method and assembled using the SeqMan program (Lasergene software v7.1, DNAstar, Inc., Madison, WI, USA). To construct the infectious clone, the pTVT vector was derived from pLaSota-DM plasmid using TVT-F and TVT-R primers. The TJ-WT genome was inserted between the T7 promoter and the Hepatitis Delta Virus ribozyme sequence, which were retained from the pLaSota-DM plasmid. This configuration facilitates viral RNA production in cells that express T7 RNA polymerase. The genome was divided into five segments (C1, S2, S3, C4, and C5) for amplification and ligation into the pTVT vector (Table 1). A nonsense mutation at the KpnI site in the M gene was introduced using S1-R and S2-F primers to facilitate detection, and the Fcs was replaced with that of La Sota using S2-R and S3-F primers. As shown in Figure 1, homologous recombination was employed to link C1 and S2 to the pTVT vector by the Seamless Cloning and Assembly Kit (TransGen, Beijing, China). After obtaining the positive plasmid, digestion with MluI (NEB, Ipswich, MA, USA) was performed, followed by insertion of the S3 fragment. This process was repeated for the C4 and C5 fragments, resulting in the full-length infectious clone of the attenuated TJ-WT strain, named TVT-aTJ.

### 2.4. Construction of the Helper Plasmids

Based on the nucleotide sequence of the TJ-WT strain, homologous recombination primers were designed to insert the NP, P, and L protein ORF regions into the multiple cloning site of the pCI-neo mammalian expression vector. To enhance gene expression, a Kozak sequence (GCCACC) was introduced upstream of the start codon (Table 1). The L ORF was divided into two segments, L1 and L2, to ensure accurate amplification. The amplification products were then ligated into the pCI-neo vector, which has been amplified using the pCI-VF/R primers. Correct clones were confirmed by sequencing, and the resulting plasmids were designated pCI-6NP, pCI-6P, and pCI-6L.

### 2.5. Rescue and Pathogenicity of Viruses

The full-length plasmid TVT-aTJ, along with the helper plasmids pCI-6NP, pCI-6P, and pCI-6L, were co-transfected into BSR-T7 cells using Lipofectamine 2000 (Thermo Fisher, Waltham, MA, USA) in a 4:2:2:1 ratio to rescue the virus. After 4 h, the medium was replaced with Dulbecco’s modified Eagle’s medium (DMEM) supplemented with 2% fetal bovine serum (FBS, Gibco, Waltham, MA, USA) and 1 µg/mL Tosyl phenylalanyl chloromethyl ketone (TPCK)-trypsin. Cells and supernatants were subsequently injected into 9-day-old SPF chicken embryos. The presence of the rescued virus was confirmed by hemagglutination (HA) testing according to the World Organisation for Animal Health (WOAH) manual [33]. Additionally, PPMV1-3 primers were used to detect the genetic marker, with the PCR product digested by KpnI to verify its presence. PPMV1–4 primers were employed to sequence the Fcs to confirm the introduced mutations [32]. The pathogenicity of the rescued virus was assessed by the standard mean death time (MDT) and intracerebral pathogenicity index (ICPI) tests, as previously described [33].

### 2.6. Virus Titration and Growth Curve

Viral titers were determined using the 50% embryo infectious dose (EID_50_) method in 9-day-old chicken embryos [33] employing the fifth-generation virus isolated from chicken embryo allantoic fluid. Additionally, viral growth kinetics were evaluated using the 50% tissue culture infectious dose (TCID_50_) assay in BHK21 cells. Briefly, 100 μL of ten-fold serial dilutions of the virus were added to a 96-well plate containing a monolayer of BHK21 cells, with each dilution replicated six times. After 1 h of incubation, the supernatant was replaced with 100 µL of fresh DMEM containing 2% FBS and 1 µg/mL TPCK-trypsin. The cells were incubated for 60 h, and cytopathic effects (CPE) were observed to assess viral infection. TCID_50_ values were calculated using the Reed–Muench method [34]. Based on the calculated virus titers, BHK21 cell monolayers were infected with virus at a multiplicity of infection (MOI) of 1. Supernatants were collected every 12 h for further TCID_50_ determination.

### 2.7. Immunization and Challenge

Four-month-old pigeons, with no prior exposure to NDV or vaccination, were sourced from a farm in Beijing, China. The pigeons were randomly divided into two groups (*n* = 10 per group). One group was immunized with the aTJ strain (10^6^ EID_50_) via eyedrop and intranasal routes, while the other group was administered PBS. Both groups were housed separately in negative pressure isolators to prevent cross-contamination between groups. Twenty-one days post-primary immunization (dpi), a booster dose was administered using the same method and dosage. Blood samples were collected on 7, 14, 21, 28, and 31 dpi for hemagglutination inhibition (HI) assays to assess the NDV antibody levels. On 31 dpi, pigeons were challenged with variant NDV genotype VI (TJ-WT) at a dose of 10^6^ EID_50_ via intramuscular injection. Clinical signs and mortality were monitored daily for 14 days post-challenge (dpc) [35,36]. Cloacal swabs were collected on 5 and 10 dpc for virus isolation. At the end of the experiment, all pigeons were euthanized with carbon dioxide.

### 2.8. HI Assay

Serum samples were collected weekly from each group. To eliminate nonspecific agglutination, sera were incubated with 20% chicken red blood cells (RBCs) at 4 °C for 1 h, followed by centrifugation at 800× *g* for five minutes. The supernatants were collected and saved at −80 °C. HI antibody titers against NDV were determined using standard procedures [33]. Both aTJ and La Sota strains were used as 4 HA unit antigens to measure the HI titers, enabling a comparison of the immune responses elicited by the two strains.

### 2.9. Virus Isolation

Cloacal swabs were collected from each pigeon in each group at 5 and 10 dpc to evaluate viral shedding. Swabs were immersed in 1 mL of phosphate-buffered saline (PBS) containing penicillin (10,000 units/mL), streptomycin (10 mg/mL), and gentamicin (250 µg/mL) and incubated at 37 °C for 2 h. Subsequently, 200 μL supernatant was inoculated into 9-day-old chicken embryos. The virus presence was confirmed 4 days later via the HA assay.

### 2.10. Statistical Analysis

All statistical analyses were conducted using GraphPad Prism Software 9.5. HI titers were presented as the median and interquartile range (IQR) and were analyzed using the nonparametric Mann–Whitney *U* test. Morbidity and mortality rates between groups were compared using the chi-square test and Fisher’s exact test. Statistical significance was defined as *p* < 0.05, with levels of significance indicated as * *p* < 0.05, ** *p* < 0.01, *** *p* < 0.001, and **** *p* < 0.0001.

## 3. Results

### 3.1. Generation of Recombinant aTJ Virus

**Figure 2:**
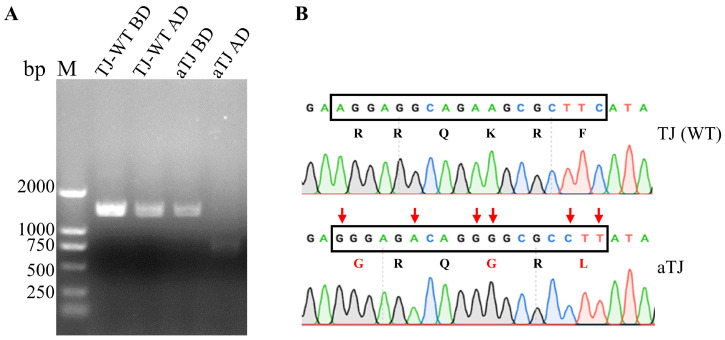
Detection of genetic marker and F protein cleavage site mutations in the recombinant aTJ virus. (**A**) Agarose gel electrophoresis of PCR products before digestion (BD) and after digestion (AD) with KpnI. Lane M represented the molecular marker. TJ-WT represented the wild-type strain, and aTJ represented the recombinant virus strain. The expected band pattern confirmed the successful digestion at the KpnI site in the recombinant virus. (**B**) Sequencing chromatogram showing the Fcs. TJ-WT exhibited the virulent cleavage site _112_RRQKRF_117_, whereas the recombinant aTJ strain had the attenuated cleavage site _112_GRQGRL_117_. Red arrows marked the nucleotide changes, and the corresponding amino acid substitutions (in red) represented the introduction of the avirulent cleavage site.

The complete genome of strain TJ-WT was amplified using eleven primer pairs, generating overlapping fragments that were subsequently assembled to produce the full genome sequence (Figure S1). The assembled genome was determined to be 15,192 nucleotides, consistent with other NDV genotype VI strains. The Fcs was identified as _112_RRQKRF_117_, indicating a virulent phenotype. A full-length infectious clone of the attenuated aTJ strain was constructed by assembling five overlapping cDNA fragments (Figure S2) replicating the TJ-WT genome, except for a silent mutation introduced at the KpnI site in the M gene for detection purposes (Figure 1). The genetic marker was verified by KpnI digestion of the PCR product amplified with PPMV1-3 primers. To attenuate the virus, the wild-type Fcs was replaced with the avirulent _112_GRQGRL_117_, commonly found in La Sota strains. This substitution resulted in three amino acid changes (positions 112, 115, and 117), confirmed through sequencing (Figure 2B). The recombinant aTJ virus was successfully rescued by co-transfecting BSR T7/5 cells with the full-length plasmid (TVT-aTJ) and helper plasmids encoding NP, P, and L proteins. After 48 h, the cells and supernatants were inoculated into 9-day-old embryonated chicken eggs, and the presence of the rescued virus was confirmed by HA testing, which showed an initial HA titer of 3 log2.

### 3.2. Biological Characterization of aTJ

**Figure 3:**
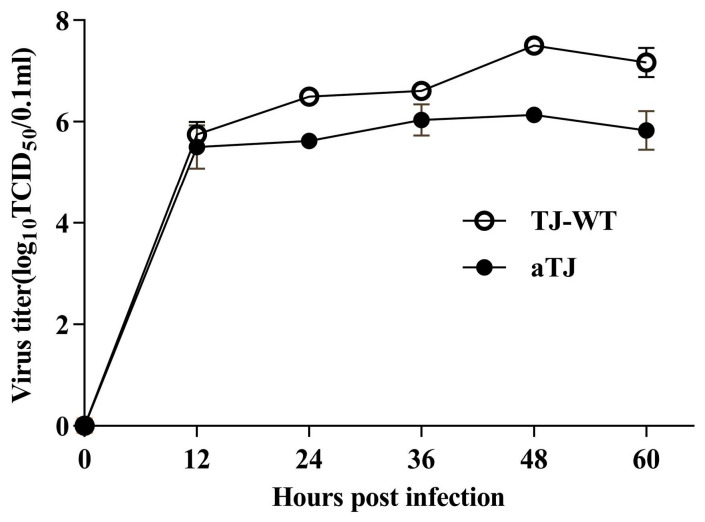
Viral growth kinetics in BHK21 cells. Cells were infected with each virus at a multiplicity of infection (MOI) of 1. Supernatants were collected every 12 h, and viral titers were determined using TCID_50_ assays. Data are expressed as the mean ± standard deviation from three independent experiments.

The virulence and replication characteristics of the fifth-generation aTJ virus, propagated in chicken embryo allantoic fluid, was assessed to confirm the attenuation and replication properties. Compared to the parental TJ-WT strain, aTJ exhibited significant attenuation, with the ICPI reduced to 0.2 and a MDT of 146 h (Table 2), indicating its classification as a lentogenic strain. The viral growth kinetics were assessed in BHK21 cells at a MOI of 1 (Figure 3). Both the aTJ and TJ-WT strains reached peak titers at 48 h post-infection, with aTJ displaying slightly lower overall titers compared to TJ-WT. These results confirm that aTJ retains efficient replication similar to the parental strain.

**Table 2:** Virulence analysis results.

Strains	lgTCID_50_^1^ (0.1 mL)	lgEID_50_^2^ (0.1 mL)	MDT ^3^ (h)	ICPI ^4^
aTJ	−8.63	−9.00	146	0.20
TJ-WT	−8.17	−8.38	62	1.19

^1^ lgTCID_50_, logarithm base 10 of 50% tissue infectious dose.^2^ lgEID_50_, logarithm base 10 of 50% egg culture infective dose.^3^ MDT, mean death time.^4^ ICPI, intracerebral pathogenicity index.

### 3.3. Antibody Response

**Figure 4:**
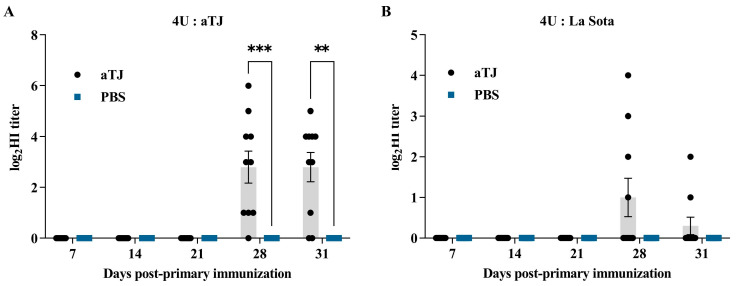
Hemagglutination inhibition (HI) titers post-immunization. Serum samples were collected at 7, 14, 21, 28, and 31 days post-primary immunization (dpi), and HI titers were measured using aTJ (**A**) and La Sota (**B**) as 4 HA unit antigens. Antibody titers are shown as log_2_ values, and data are presented as the median and interquartile range (IQR) (*n* = 10/group). A booster immunization was given on 21 dpi. Statistical comparisons were made using the nonparametric Mann–Whitney *U* test. Significance levels: ** *p* < 0.01 and *** *p* < 0.001. Values without asterisks indicate no statistically significant difference.

Serum samples were collected on 7, 14, 21, 28, and 31 dpi, and HI titers were measured using both the aTJ and La Sota strains as antigens (Figure 4). Following the booster immunization on 21 dpi, the aTJ vaccine group exhibited a significant increase in antibody titers. In the HI assays using aTJ as the antigen (Figure 4A), the aTJ group showed a sudden rise in antibody titers on 28 dpi, with the median titer slightly decreasing on 31 dpi, while the PBS control group remained negative throughout the experiment. The antibody titers in the aTJ group were significantly higher than those in the PBS group. Similarly, when La Sota was used as the antigen (Figure 4B), some pigeons in the aTJ group were seroconverted by 28 dpi, though the median titer also declined slightly on 31 dpi. Notably, the antibody titers detected using aTJ as the antigen were higher than those observed with La Sota, indicating that the aTJ vaccine elicited a robust, strain-specific immune response.

### 3.4. Clinical Signs in Vaccinated Pigeon After Challenge

**Figure 5:**
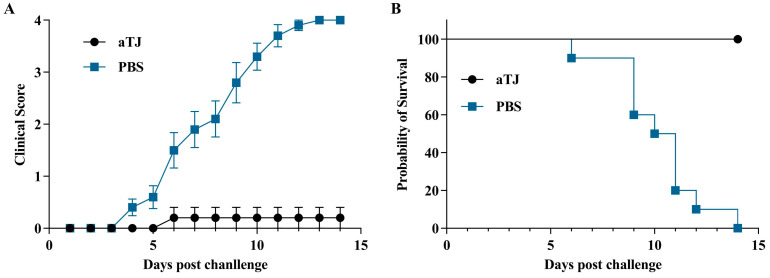
Clinical scores and survival curves post-challenge with the TJ-WT strain. (**A**) Clinical scores for each group of inactivated vaccines. Clinical scores for each group were expressed as the mean ± standard error of the mean (SEM). The clinical symptoms were assessed based on the scoring criteria: 0 points for normal state; 1 point for depression or neck shrinkage; 2 points for drooping wings, torticollis, or ataxia; 3 points for general paralysis or collapse; and 4 points for death. (**B**) Survival curves for the groups after the challenge. Mortality rates were 100% in the PBS group, while mortality in the aTJ groups was 0%.

Each group was challenged with the TJ-WT strain on day 10 post-boost immunization (31 dpi), with the clinical signs monitored daily for 14 days. Clinical symptoms observed in pigeons are shown in Figure S3, and detailed clinical observations for each group throughout the experiment are provided in Table S1. Clinical scores and survival curves are shown in Figure 5. In the PBS group, clinical signs appeared at 4 dpc, including lethargy, ruffled feathers, and yellow-green or white, loose feces. Symptoms worsened, and one pigeon died on 6 dpc. From 9 dpc, mortality increased steadily, ultimately reaching 100% by 13 dpc, with a peak clinical score of 4.0. In contrast, in the aTJ group, only one pigeon exhibited mild clinical symptoms from 6 dpc, with a stable clinical score of 0.2, and no pigeons succumbed to the infection, resulting in a morbidity rate of 10% (1/10) and a mortality rate of 0% (0/10). These results demonstrate that the aTJ vaccine effectively protects pigeons from the lethal challenge of PPMV-1.

### 3.5. Reduction in Virulent NDV Shedding

Virus shedding was evaluated through cloacal swabs collected at 5 and 10 dpc (Table 3). At 5 dpc, virus shedding was detected in 100% of the pigeons in the PBS control group while the aTJ group exhibited a shedding rate of 30% (3/10). By 10 dpc, virus shedding in the aTJ group decreased to 10% (1/10), while the PBS group maintained consistent shedding at 100%. Notably, the three pigeons in the aTJ group that tested positive for virus shedding at 5 dpc had HI antibody titers of less than 1 log2 at 31 dpi. Additionally, compared to the PBS group, the aTJ groups displayed significantly lower morbidity (10%, 1/10, *p* = 0.0001) and mortality rates (0%, 0/10, *p* < 0.0001). These results suggest that the live attenuated aTJ vaccine effectively reduces virus shedding while providing strong protection against morbidity and mortality.

## 4. Discussion

PPMV-1 was first detected in Hong Kong in 1985 and has since become a major pathogen impacting pigeon breeding in the region [10]. Inactivated and live attenuated La Sota strain vaccines are commonly used for PPMV-1 prevention. However, sporadic outbreaks of Newcastle disease continue, suggesting suboptimal vaccine protection and highlighting the need for improved strategies. PPMV-1 is an antigenically distinct genotype of NDV, classified as genotype VI. In recent years, the predominant circulating PPMV-1 strains have been subtypes VI.2.1.1.2.1 and VI.2.1.1.2.2, with VI.2.1.1.2.2 gradually emerging as the dominant strain [37,38]. The PPMV-1 strain TJ (WT) was isolated from a pigeon loft in Tianjin that had been vaccinated with the La Sota vaccine. This strain exhibited an antigenic similarity coefficient of 0.13 relative to La Sota, indicating significant antigenic divergence [30]. A lot of research has demonstrated that vaccines with a close antigenic relationship to the currently circulating strains may offer better protection compared to commercial vaccines [20,39,40]. Furthermore, live attenuated NDV vaccines are particularly well suited for mass production and are routinely utilized for large-scale vaccination via spray or drinking water administration, which significantly streamlines the vaccination process. Therefore, selecting an attenuated live vaccine that more closely matches the antigenicity of circulating strains may represent a more effective prevention strategy.

To address the challenges associated with PPMV-1, this study applied reverse genetic technology to develop an attenuated vaccine candidate. The virulent F protein cleavage site of the TJ-WT strain (_112_RRQKRF_117_) was replaced with the attenuated cleavage site (_112_GRQGRL_117_) typically present in the La Sota strain. This modification resulted in a significant reduction in virulence, as indicated by the ICPI of 0.2 and MDT of 146 h, consistent with an attenuated phenotype. The findings confirm that targeted mutations at the Fcs can effectively reduce virulence [20,29], thereby supporting the potential of aTJ as a viable vaccine candidate with diminished pathogenicity while retaining its immunogenic properties.

Further evaluation of the aTJ strain’s protective efficacy as a live attenuated vaccine was conducted. The HI antibody titer, a key measure of humoral immunity, was undetectable in pigeons until 21 dpi, consistent with prior findings of delayed and weaker antibody responses to PPMV-1 compared to chickens [41]. Although no HI titers were detected until 21 dpi, this does not imply a lack of immune response. It is possible that cellular immunity, stimulated via eyedrop and intranasal routes, may have enhanced the humoral immune response upon booster immunization. Studies have shown that PPMV-1-infected pigeons exhibit elevated IFN-γ levels, indicating a cell-mediated immune response [42,43,44,45,46]. This cellular immunity may have primed the pigeons for a stronger antibody response after the second immunization. Seroconversion (HI antibody titer) was observed on day 7 after the booster immunization, further confirming that the attenuated vaccine is capable of replicating in pigeons and eliciting a host immune response [47]. However, the antibody levels following the second immunization varied among individuals, with some pigeons rapidly producing antibody titers as high as 6 log2, while one pigeon remained at 0 log2. This variability is likely attributable to differences in animal types and individual responses [41]. Using aTJ and La Sota as four-unit antigens to measure pigeon HI antibody levels revealed a difference of up to 4 log2, indicating significant antigenic variations between the two strains [30]. HI titers against homologous antigens were significantly higher in different vaccine groups compared to heterologous antigens. Although La Sota is often used in HI tests for immunized animals, its titers may not fully correlate with protection against specific pathogens. To ensure accurate correlations between HI titers and protective efficacy, the challenge isolate should be used as the working antigen.

The virulent TJ-WT strain was used to challenge the pigeons on the 31 dpi. In the PBS control group, all pigeons exhibited symptoms from 4 dpc, with 100% incidence and mortality. In contrast, only one pigeon in the aTJ group showed mild symptoms, such as drooping wings, but did not worsen, and no deaths occurred. The incidence rate in the aTJ group was 10%, with a mortality rate of 0%. Antigen-matched vaccines have shown effective protection against viral challenges [19,20,21]. Vaccination strategies are effective in reducing the severity of clinical manifestations and mortality in poultry infected with NDV, though they do not entirely prevent infection or subsequent viral shedding. NDV primarily spreads through the fecal–oral route [42], making the reduction of viral shedding vital for controlling transmission among poultry populations [48]. As a result, viral shedding is a key indicator of a vaccine’s efficacy. The viral shedding rate in the PBS group was 100% on both the 5 and 10 dpc. In contrast, only three pigeons in the aTJ group with HI titers below 1 log2 were shedding the virus on 5 dpc, and only one symptomatic pigeon continued shedding on 10 dpc. This outcome underscores the aTJ vaccine’s superior protective efficacy, showing a strong correlation between higher HI titers and enhanced immunological protection [11,49,50]. Although the aTJ vaccine demonstrated significant short-term efficacy in this study, the duration of vaccine-induced immunity remains a critical factor for the long-term control of PPMV-1. Future studies will extend the observation period to evaluate the persistence of HI titers, reduction in virus shedding, and the overall durability of immune protection over time. Such studies will provide a more comprehensive understanding of the vaccine’s potential for sustained immunity and its practical application in pigeon breeding programs.

## 5. Conclusions

This study demonstrated the strong potential of the aTJ strain, developed through reverse genetic modification, as a live attenuated vaccine for PPMV-1 in pigeons. The vaccine effectively reduced virulence while maintaining immunogenicity, leading to a significant increase in HI titers after booster immunization, as well as reduced viral shedding and complete protection against mortality. These findings emphasize the importance of antigenic matching in vaccine design and provide a solid foundation for future research to evaluate the vaccine’s long-term protective efficacy and practical applications in pigeon breeding programs.

## Figures and Tables

**Table 1:** Primers used for virus rescue and identification.

Primer Names	Primer Sequence (5′→3′)
C1-F	CGACTCACTATAGGACCAAACAGAGAATCCGTGAGTTA
C1-R	CC*gGTaCC*TTGTAGGACGATC
S2-F	GTTAGCATTCCCGATCGTCCTACAA*GGtACc*GGTG
S2-R	AGGAGGTGGAGATGCCATGCCGACCC*ACGCGT*TATaAGgCGCccCTGTCTCCc
S3-F	GAgGGAGaCAGggGCGCCTTATAG
S3-R	GTGGAGATGCCATGCCGACCC*ACGCGT*GGAGCTCGCCATTTCCTACCCG
C4-F	ACGGGTAGGAAATGGCGAG
C4-R	AGGAGGTGGAGATGCCATGCCGACCC*ACGCGT*CTGTTCCGGGCATAGTCTG
C5-F	CTCGCTGACGCTAGCAG
C5-R	GAGGAGGTGGAGATGCCATGCCGACCCACCAAACAAAGATTTGGTGAATG
TVT-F	GGGTCGGCATGGCATCTCCAC
TVT-R	TGGTCCTATAGTGAGTCGTATTAATTTCGCGGG
6NP-F	CACTATAGGCTAGCCTCGAGGCCACCATGTCGTCCGTCTTTG
6NP-R	CTCTAGAGGTACCACGCGTTCAGTACCCCCAGTCGGTG
6P-F	CACTATAGGCTAGCCTCGAGGCCACCATGGCAACTTTTACTGATGCTG
6P-R	TCTAGAGGTACCACGCGTTCAACCATTCAGTGCAAGGC
6L1-F	CACTATAGGCTAGCCTCGAGGCCACCATGGCGAGCTCCGGTCCTGAAAG
6L1-R	GCTAGCGTCAGCGAGCACATAGC
6L2-F	GTCTGCTATGTGCTCGCTG
6L2-R	CTCTAGAGGTACCACGCGTTTAGGAGTCATTGTTACTGTAATATCCCTTTG
pCI-V-F	ACGCGTGGTACCTCTAGAGTC
pCI-V-R	CTCGAGGCTAGCCTATAGTGAGTC
PPMV1-3F ^1^	TCAAAGCAGACATCCTCCA
PPMV1-3R ^1^	AAATGTMACTTTCTTTCCCCTCT
PPMV1-4F ^2^	ATGTCACTATTGAYGTGGAGGTA
PPMV1-4R ^2^	GGACAAGTGCTGAGGCAAAYC

^1^ Primers were used to detect genetic markers. ^2^ Primers were used to detect the Fcs. Lowercase letters represented mutated bases, italicized letters indicated restriction enzyme recognition sites, and underlined text denoted the Kozak sequence.

**Table 3:** Frequency of isolation of the challenge virus in different vaccine groups.

Groups	Number	Virus Shedding		Morbidity	Mortality
		5 dpc	10 dpc		
aTJ	10	3/10	1/10	1/10	0/10
PBS	10	10/10	5/5	10/10 ***	10/10 ****

The number “n/m” indicates the number of positives detected and the total number of related samples. “dpc” represents days post-challenge. Statistical significance was analyzed using the chi-square (and Fisher’s exact) test with GraphPad Prism software 9.5. *** *p* < 0.001, and **** *p* < 0.0001.

## Data Availability

The data presented in this study are available on request from the corresponding author.

## Supplementary Materials

The following supporting information can be downloaded at https://www.mdpi.com/article/10.3390/vaccines12121304/s1: Figure S1: PCR amplification of the TJ-WT genome segments; Figure S2: Construction of the full-length infectious clone. Figure S3: Clinical symptoms with corresponding scores. Table S1. Detailed clinical observations of pigeons in each group after the challenge.
