# Supplementary material for: Live Attenuated aTJ Vaccine Effectively Protects Pigeons Against Homologous PPMV-1 Challenge
Source: Vaccines (Basel). 2024 Nov 22;12(12):1304. doi: 10.3390/vaccines12121304 (PMC11680285; doi:10.3390/vaccines12121304)
Supplement: Supplementary file 1 [file vaccines-12-01304-s001.zip › Supplementary_Figures.pdf]

Supplementary Figures legends

Figure S1: PCR amplification of TJ-WT genome segments. The PCR amplification of the TJ-WT genome was performed using 11 pairs of primers. Lanes 1–11 represent the amplified segments of the TJ20 strain genome.

Figure S2: Construction of the full-length infectious clone. Lanes C1, S2, S3, C4, and C5 correspond to the five amplified genome segments inserted into the pTVT vector. The bands in each lane correspond to the expected sizes of the segments.

Figure S3. Clinical symptoms with corresponding scores. (**A**) Healthy pigeon, score 0. (**B**) Depression, ruffled feathers or neck shrinkage, score 1. (**C**) Drooping wings, score 2. (**D**) Twisted neck, neurological symptoms, score 3. (**E**) Paralysis, score 3. (**F**) Death, score 4.
