# Supplementary material for: Live Attenuated aTJ Vaccine Effectively Protects Pigeons Against Homologous PPMV-1 Challenge
Source: Vaccines (Basel). 2024 Nov 22;12(12):1304. doi: 10.3390/vaccines12121304 (PMC11680285; doi:10.3390/vaccines12121304)
Supplement: Supplementary file 1 [file vaccines-12-01304-s001.zip › Table S1.pdf]

Table S1. Detailed clinical observations of pigeons in each group after challenge.

Groups	Subjects	Days post challenge(dpc)													
		**1**	**2**	**3**	**4**	**5**	**6**	**7**	**8**	**9**	**10**	**11**	**12**	**13**	**14**
**PBS**	1	health	health	health	ruffled feathers	ruffled feathers, depression	ruffled feathers, depression	ruffled feathers, depression	ruffled feathers, depression	ruffled feathers, depression	death	death	death	death	death
	2	health	health	health	health	health	ruffled feathers	ruffled feathers	ruffled feathers	death	death	death	death	death	death
	3	health	health	health	health	health	health	drooping wings	drooping wings	drooping wings	drooping wings	death	death	death	death
	4	health	health	health	health	health	drooping wings	drooping wings	drooping wings, twisted neck	drooping wings, twisted neck	drooping wings, twisted neck	drooping wings, twisted neck	death	death	death
	5	health	health	health	health	health	ruffled feathers	ruffled feathers	paralysis	death	death	death	death	death	death
	6	health	health	health	health	depression, drooping wings	depression, drooping wings	paralysis	paralysis	paralysis, twisted neck	paralysis, twisted neck	death	death	death	death
	7	health	health	health	ruffled feathers	ruffled feathers	death	death	death	death	death	death	death	death	death
	8	health	health	health	ruffled feathers	ruffled feathers	drooping wings	paralysis	paralysis	paralysis	paralysis	paralysis	paralysis	death	death
	9	health	health	health	ruffled feathers,	ruffled feathers,	ruffled feathers,	ruffled feathers,	ruffled feathers,	ruffled feathers,	paralysis	death	death	death	death

					depression	depression	depression	depression	depression	depression					
	10	health	health	health	health	health	depression	ruffled feathers, depression	ruffled feathers, depression	death	death	death	death	death	death
**aTJ**	1	health	health	health	health	health	ruffled feathers, drooping wings	ruffled feathers, drooping wings	drooping wings	drooping wings	drooping wings	drooping wings	drooping wings	drooping wings	drooping wings
	2	health	health	health	health	health	health	health	health	health	health	health	health	health	health
	3	health	health	health	health	health	health	health	health	health	health	health	health	health	health
	4	health	health	health	health	health	health	health	health	health	health	health	health	health	health
	5	health	health	health	health	health	health	health	health	health	health	health	health	health	health
	6	health	health	health	health	health	health	health	health	health	health	health	health	health	health
	7	health	health	health	health	health	health	health	health	health	health	health	health	health	health
	8	health	health	health	health	health	health	health	health	health	health	health	health	health	health
	9	health	health	health	health	health	health	health	health	health	health	health	health	health	health
	10	health	health	health	health	health	health	health	health	health	health	health	health	health	health
