# Supplementary figures and images for: Live Attenuated aTJ Vaccine Effectively Protects Pigeons Against Homologous PPMV-1 Challenge
Source: Vaccines (Basel). 2024 Nov 22;12(12):1304. doi: 10.3390/vaccines12121304 (PMC11680285; doi:10.3390/vaccines12121304)

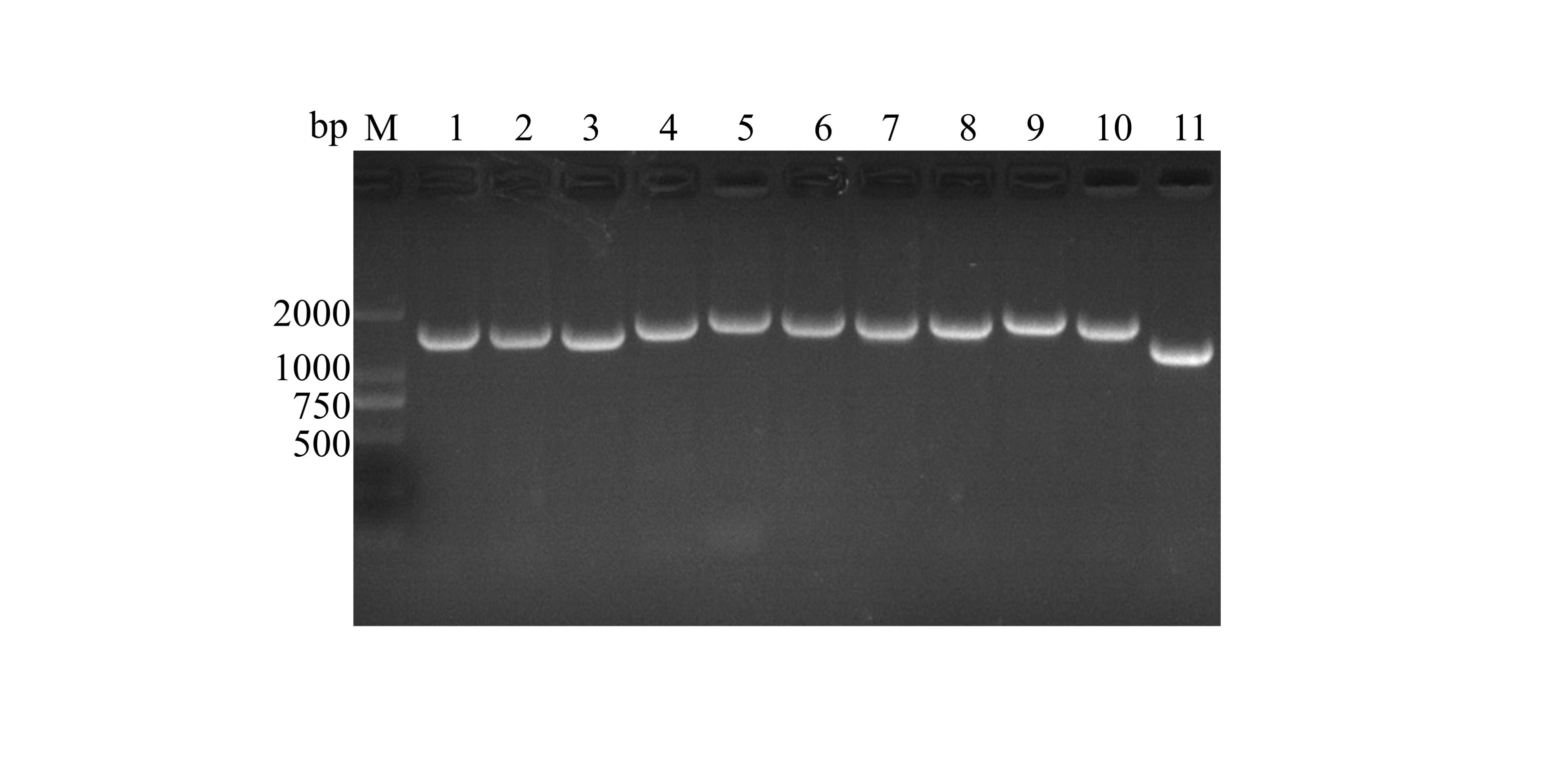

Supplement: Supplementary file 1 [file vaccines-12-01304-s001.zip › Figure S1.jpg]

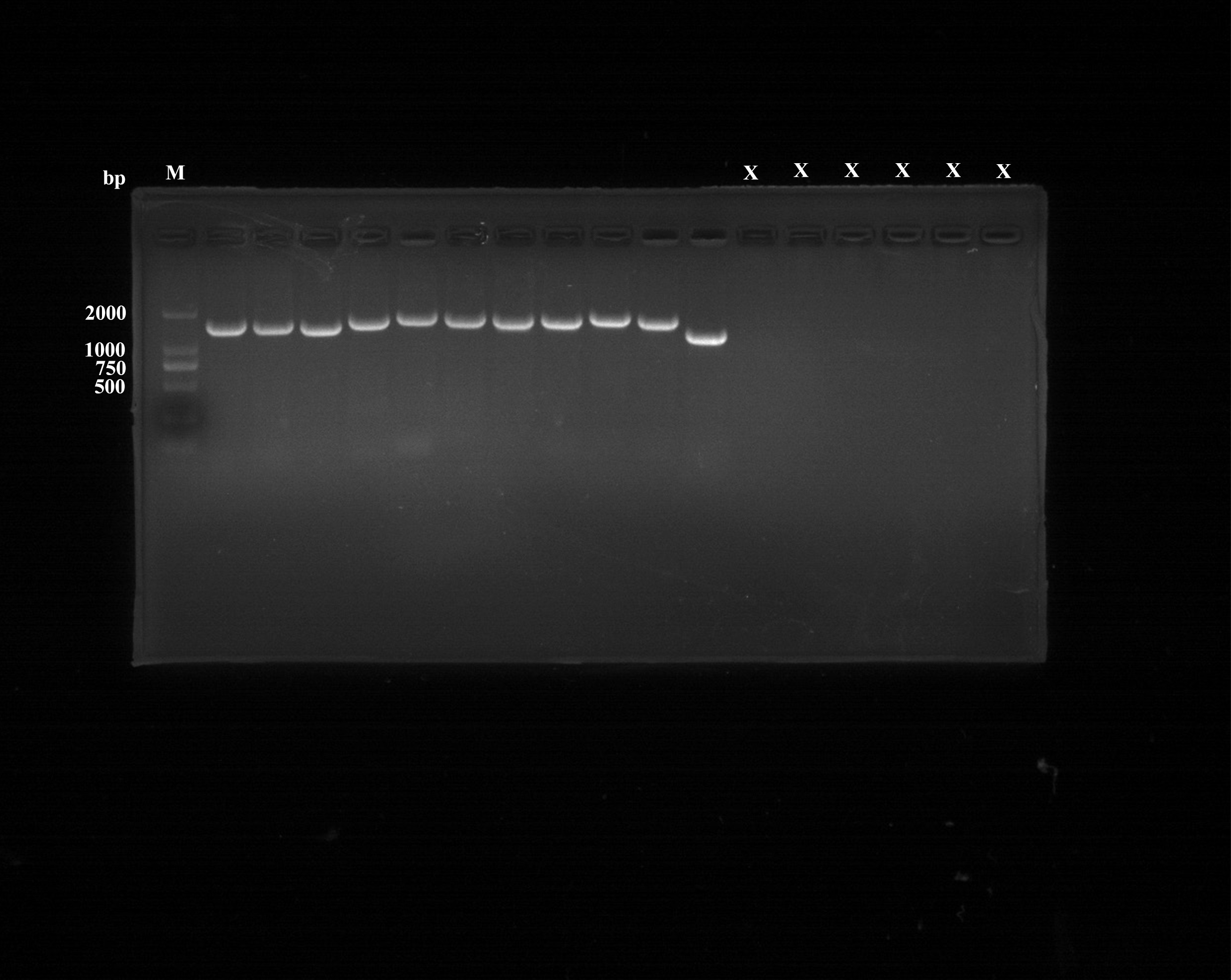

Supplement: Supplementary file 1 [file vaccines-12-01304-s001.zip › Figure S1.png]

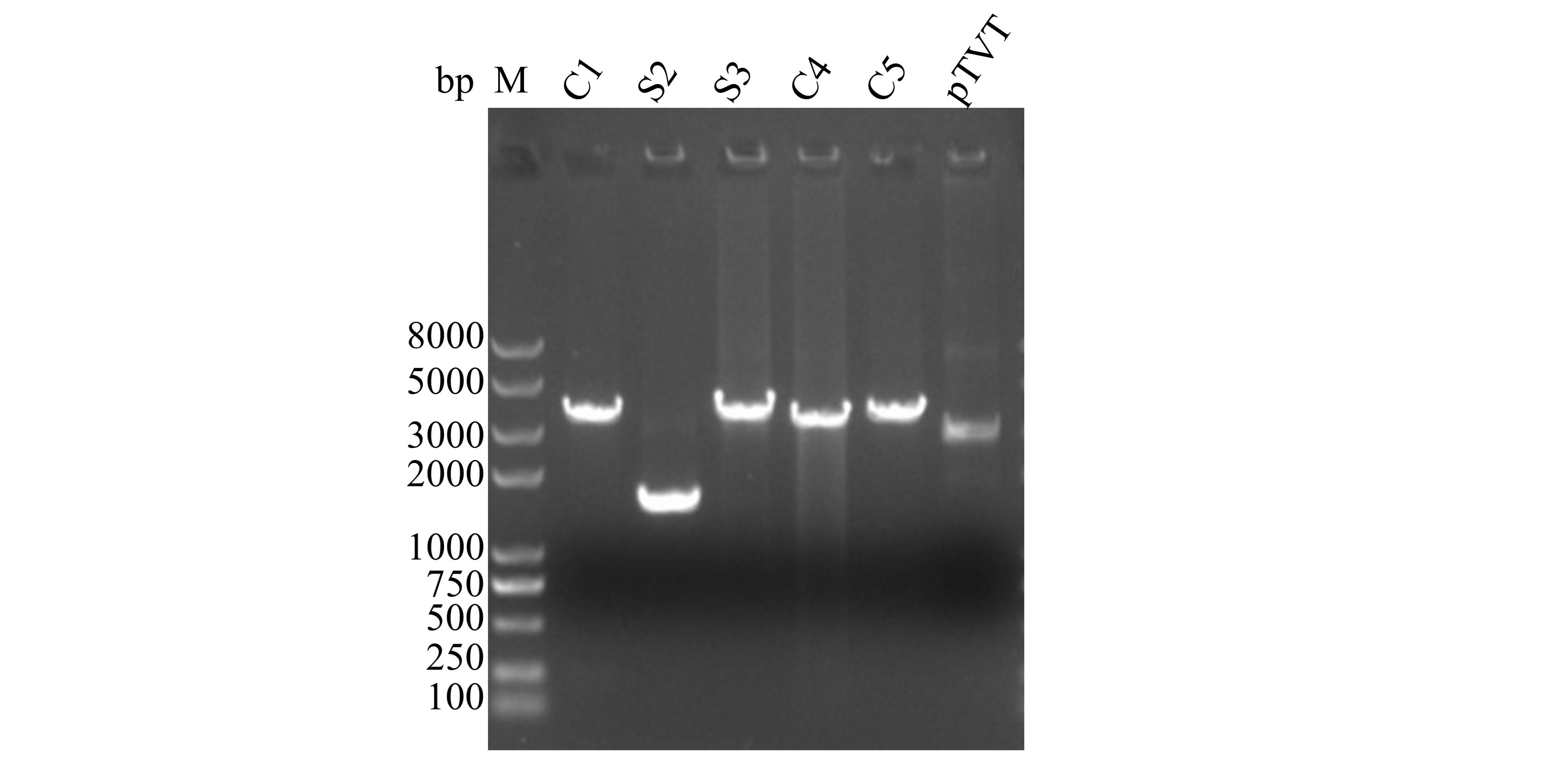

Supplement: Supplementary file 1 [file vaccines-12-01304-s001.zip › Figure S2.jpg]

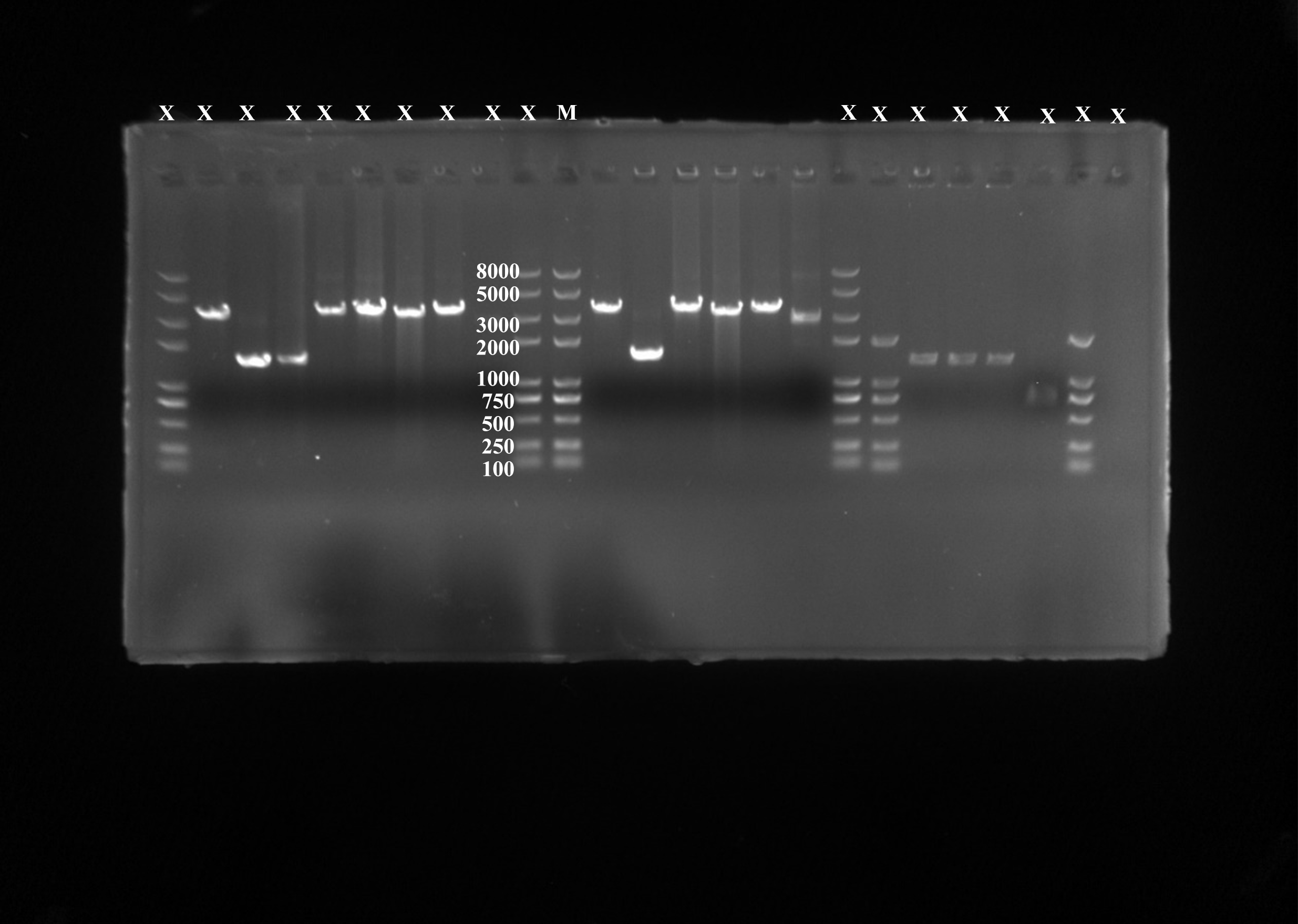

Supplement: Supplementary file 1 [file vaccines-12-01304-s001.zip › Figure S2.png]

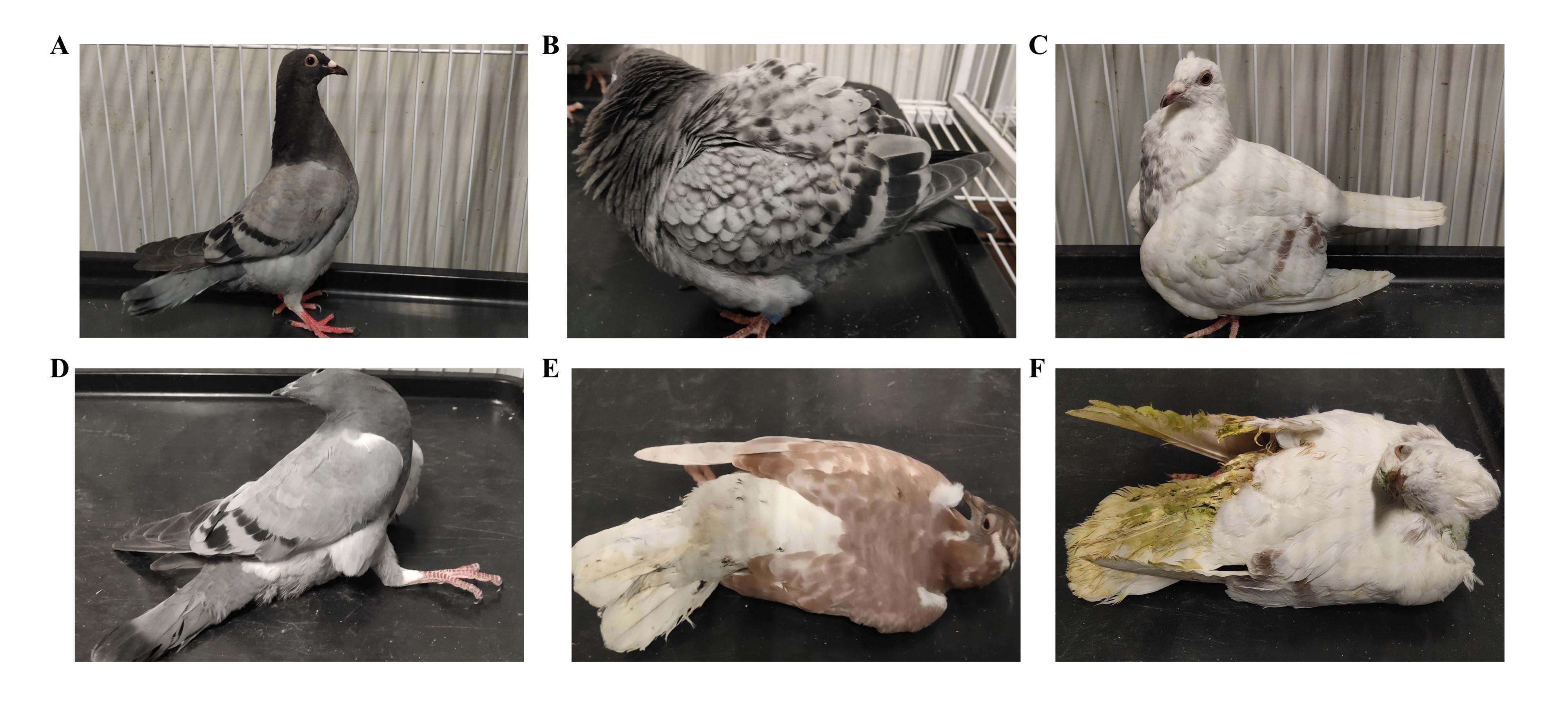

Supplement: Supplementary file 1 [file vaccines-12-01304-s001.zip › Figure S3.jpg]
